# Heterogeneity of cancer-associated fibroblasts and tumor-promoting roles in head and neck squamous cell carcinoma

**DOI:** 10.3389/fmolb.2024.1340024

**Published:** 2024-06-20

**Authors:** Imane El Herch, Stian Tornaas, Harsh Nitin Dongre, Daniela Elena Costea

**Affiliations:** ^1^ University of Lyon, Université Claude Bernard Lyon 1, INSERM 1052, CNRS 5286, Centre Léon Bérard, Centre de Recherche en Cancérologie de Lyon, Lyon, France; ^2^ Gade Laboratory for Pathology, Department of Clinical Medicine, Faculty of Medicine and Dentistry, University of Bergen, Bergen, Norway; ^3^ Centre for Cancer Biomarkers (CCBIO), Faculty of Medicine and Dentistry, University of Bergen, Bergen, Norway

**Keywords:** cancer-associated fibroblasts, head and neck squamous cell carcinoma, tumor microenvironment, heterogeneity, therapeutic targeting

## Abstract

Tumor microenvironment (TME) in head and neck squamous cell carcinoma (HNSCC) has a major influence on disease progression and therapy response. One of the predominant stromal cell types in the TME of HNSCC is cancer-associated fibroblasts (CAF). CAF constitute a diverse cell population and we are only at the beginning of characterizing and understanding the functions of various CAF subsets. CAF have been shown to interact with tumor cells and other components of the TME to shape mainly a favourable microenvironment for HNSCC progression, although some studies report existence of tumor-restraining CAF subtypes. The numerous pathways used by CAF to promote tumorigenesis may represent potential therapeutic targets. This review summarizes current knowledge on the origins, subtypes and mechanisms employed by CAF in HNSCC. The aim is to contribute to the understanding on how CAF actively influence the TME and modulate different immune cell types, as well as cancer cells, to establish a conducive setting for cancer growth. Although CAF are currently a promising therapeutic target for the treatment of other types of cancer, there is no significant therapeutic advancement in HNSCC.

## Introduction

**FIGURE 1:**
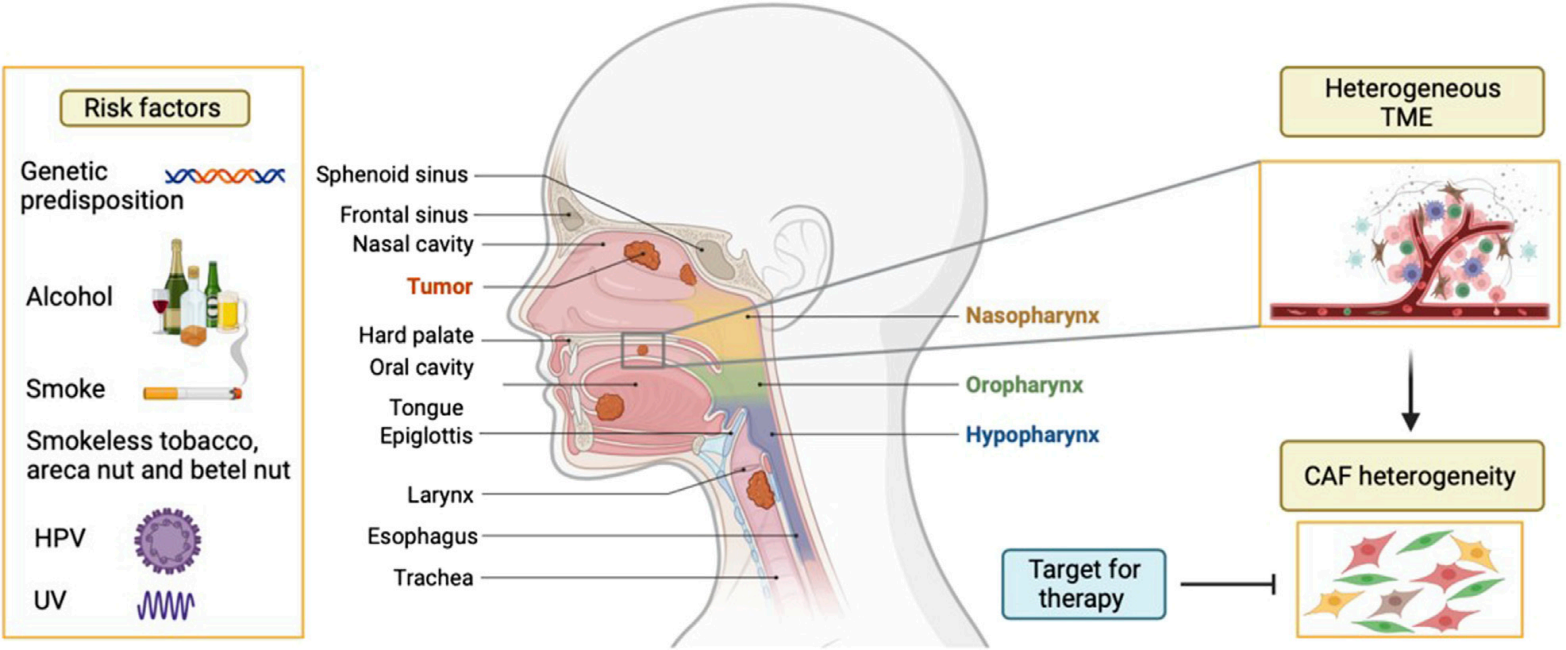
Etiology of head and neck cancers. Head and neck cancers are localized in the upper aerodigestive tract and mainly arise in the stratified squamous epithelium, hence called head and neck squamous cell carcinoma (HNSCC). Different risk factors are known for the development of HNSCC, such as genetic makeup, alcohol, smoked and smokeless tobacco, UV, areca nut, and more recently HPV for oropharynx. HNSCC is often characterised by a heterogeneous tumour microenvironment (TME), particularly due to presence of different subtypes of cancer associated fibroblasts (CAF). Due to various roles displayed by CAF in supporting tumor progression, this cell population is a potential therapeutic target.

Head and neck cancers (HNC) are the seventh most common cancers worldwide (Chow, 2020). HNC are highly aggressive tumors and 90% of HNC are squamous cell carcinomas (HNSCC), arising from the stratified squamous mucosal epithelium of the upper aerodigestive tract. Continuous exposure to tobacco smoking, excessive alcohol consumption, and genetic predisposition are well-known risk factors for HNSCC occurrence (Chow, 2020). Moreover, infection with high-risk human papillomaviruses (HPV) is a risk factor for oropharyngeal squamous cell carcinoma (base of tongue and tonsillar squamous cell carcinoma in particular) (Figure 1). HNSCC remains difficult to treat and requires a multidisciplinary approach. Currently, treatments for this type of cancer are mainly surgery, chemotherapy and radiotherapy singularly or in combination. Targeted therapy with epidermal growth factor receptor (EGFR) inhibitors and immune checkpoint inhibitor therapy has been introduced recently, but it is not very effective (Vermorken et al., 2008; Verma et al., 2018; Burtness et al., 2019). HNSCCs exhibit a less than 50% 5 years overall survival and severely impact the quality of life of patients. The unfavorable prognosis is attributed to poor response to treatment and development of drug resistance (Chow, 2020). Thus, there is a need to better understand biomarkers or mechanisms that can be targeted therapeutically. The contribution of tumor microenvironment (TME) to tumor growth, progression, metastasis and treatment resistance of HNSCC has been clearly shown (Dongre et al., 2022). There are several theories explaining this biological behaviour, proposing either the cancer location or the particular composition and origin of TME (Peltanova et al., 2019). The TME of HNSCCs is highly heterogeneous and is composed of different cell types. Among them, endothelial cells, pericytes, cells of mesenchymal origin (as fibroblasts and adipocytes), nerve cells and immune cells such as macrophages, dendritic cells, B and T lymphocytes, granulocytes, neutrophils, natural killer cells, mast cells, myeloid-derived suppressor cells, tumour-associated macrophages, regulatory T cells (Treg) are major players. The tumor stroma was shown to be a crucial modulator of cancer progression (Giraldo et al., 2019). In particular, the involvement of the mesenchymal TME part raised a lot of interest for its involvement in oral and head and neck epithelial tumors (Dongre et al., 2022). A dominant component of the tumor stroma is represented by fibroblasts. Cancer-associated fibroblasts (CAF), the largest cell population among non-malignant cells of the TME, are active and essential collaborators in tumorigenesis (Kalluri, 2016). Evidence about CAF’s role suggests that they promote HNSCC progression and this is correlated with more aggressive clinicopathological parameters (Dongre et al., 2022). CAF are related to many features of tumor aggressiveness, including invasion, angiogenesis, metastasis, and resistance to therapy, suggesting their multifactorial role. Understanding their numerous functions will help develop CAF-targeted strategies for treating HNSCC. More recently, the heterogeneity of this stromal population and the diverse functions of the different CAF subpopulations has begun to be depicted in different types of cancer, including HNSCC (Costea et al., 2013). Phenotypically different CAF subsets have been identified by single cell RNA sequencing (scRNA-seq) in HNSCC (Puram et al., 2017), breast (Kieffer et al., 2020), pancreatic (Ohlund et al., 2017), lung (Cords et al., 2024), and ovarian cancers (Zhao et al., 2022). In general, most studies identify two main categories of CAF, represented by myofibroblastic CAF (myCAF) and inflammatory CAF (iCAF). Obradovic et al. (2022) were able to show that the myCAF and iCAF subpopulations found in breast and pancreas cancer may be present in HNSCCs but probably with different functions.

Despite advances in characterizing CAF in HNSCC, there are significant challenges that CAF heterogeneity poses for developing CAF-targeted therapies and diagnostic tools. The lack of specific biomarkers and incomplete understanding of the molecular mechanisms driving CAF activity further complicate clinical interventions. Effective clinical strategies for CAF targeting have yet to be identified (Hanley and Thomas, 2021). Additionally, as we will highlight in this review, CAF contribute to resistance against conventional treatments, underscoring the need for new strategies to overcome this challenge. Bridging the gap between preclinical research and clinical application is essential for improving patient outcomes through personalized and combination therapies that effectively target CAF. Further characterisation of CAF heterogeneity and the elucidation of functions specific to each CAF subset might have profound implications for unravelling target subpopulations and mechanisms that can be pivotal to develop CAF-targeted strategies for more efficient treatment of HNSCC (Figure 1).

## CAF origins

**FIGURE 2:**
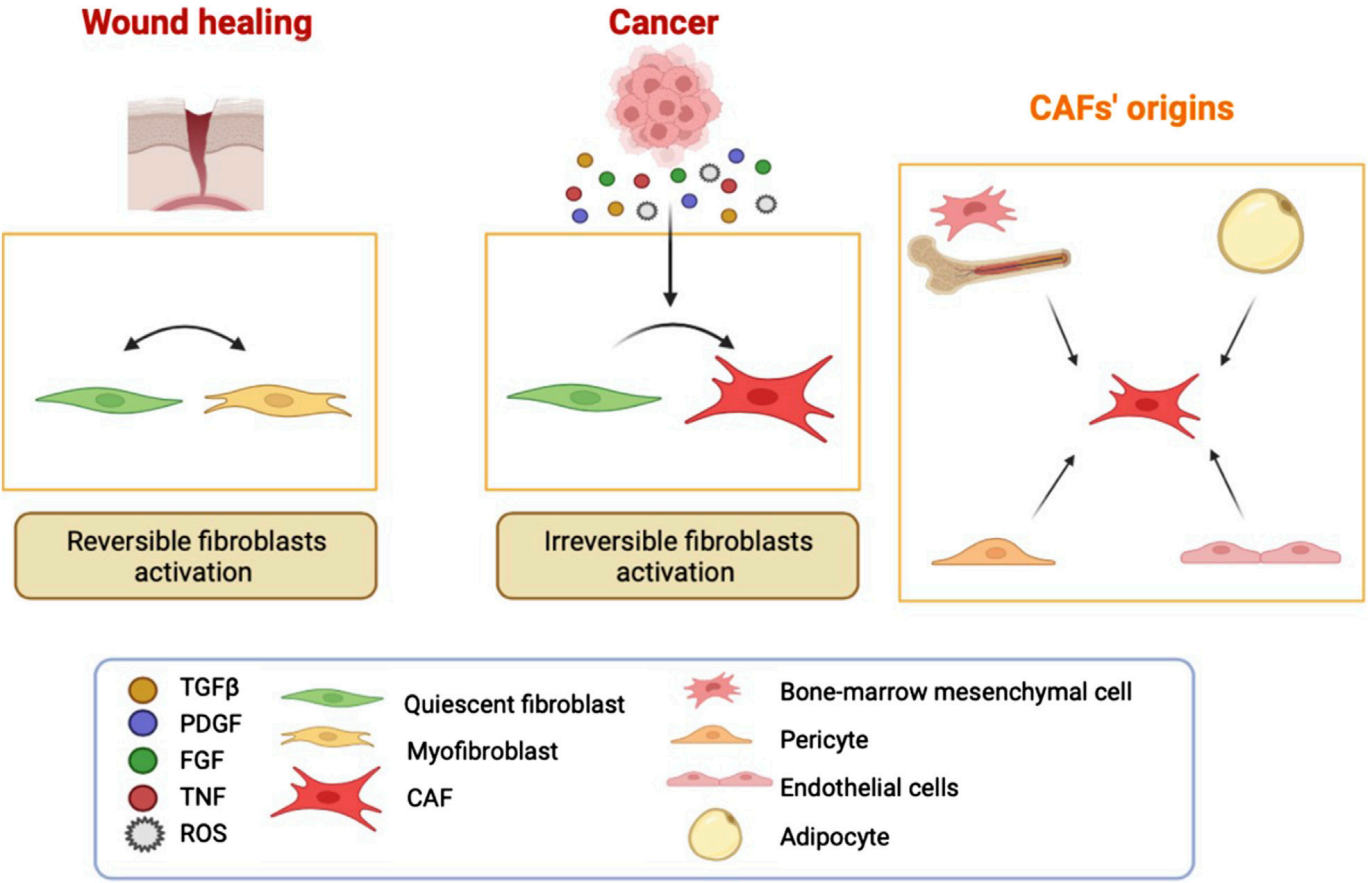
CAF have different origins. Fibroblast activation in wound healing is reversible. In cancer, there is a sustained activation due to continuous secretion of molecules by the surrounding cells such as immune and cancer cells. There are also fibroblast-independent origins for CAF. It has been shown that CAF can be derived from adipocytes, pericytes, endothelial cells, and bone-marrow mesenchymal cells.

It is considered that most of the CAF are resident fibroblasts that undergo activation due to environmental cues mirroring the activation process of a quiescent fibroblast in an injured tissue prior to repair (Kalluri, 2016). In fact, there is postulated that there are two types of fibroblast activation profiles: “reversible” and “irreversible” (Liu et al., 2019) (Figure 2). In response to tissue injury, quiescent fibroblasts are reversibly activated into myofibroblasts to facilitate repair and regeneration in a wound-healing response, while in tumor stroma the regression of myofibroblasts back to a quiescent state is impaired and their activation status is maintained mainly due to the epigenetic regulation (Costea et al., 2013). The activation of fibroblasts in many cancers is dependent on different paracrine cues such as growth factors and cytokines, *e.g.*, fibroblast growth factor (FGF), transforming growth factor-β (TGFβ), platelet-derived growth factor (PDGF), receptor tyrosine kinase signalling, tumor necrosis factor, or reactive oxygen species, released by cancer cells and the infiltrating immune cells (Zhou et al., 2022) (Figure 2).

Defining the CAF population remains challenging because there are insufficient specific markers available. CAF are frequently identified by cell morphology, spatial distribution, and absence of lineage markers (for epithelial, endothelial, and blood-born cells) (Sahai et al., 2020). Of note, none of the reported CAF markers is specific to CAF. Many of the markers used for CAF are actually highlighting different states of fibroblast activation. Among them, fibroblast-specific protein 1 (FSP) is a reliable marker for the detection of quiescent fibroblasts, as well as vimentin. Others seem to be more related to activated fibroblasts, such as alpha-smooth muscle actin (αSMA) expressed also by activated myofibroblasts in wound healing, fibroblast activating protein alpha (FAP), PDGFRα, PDGFRβ, podoplanin (PDPN), integrin alpha 11 (ITGA11), ligands belonging to the TGFβ superfamily, bone morphogenic proteins (BMPs), epidermal growth factors (EGFs), FGFs, sonic hedgehog, desmin and discoidin domain-containing receptor-2. It is likely that many functionally activated fibroblasts do not express all these putative markers simultaneously, creating thus a certain degree of heterogeneity. A reason for CAF heterogeneity can also be the different origins of CAF. Indeed, bone marrow-derived cells, adipocytes, smooth muscle cells, pericytes, and endothelial cells may (trans)differentiate to become CAF-like (Kalluri, 2016) (Figure 2). In addition, CAF heterogeneity might also be explained by their location and the different functions of the organ/area in which they reside. Recently, several lines of evidence confirm the heterogeneity and plasticity of CAF within HNSCC (Dongre et al., 2022).

## CAF heterogeneity

**FIGURE 3:**
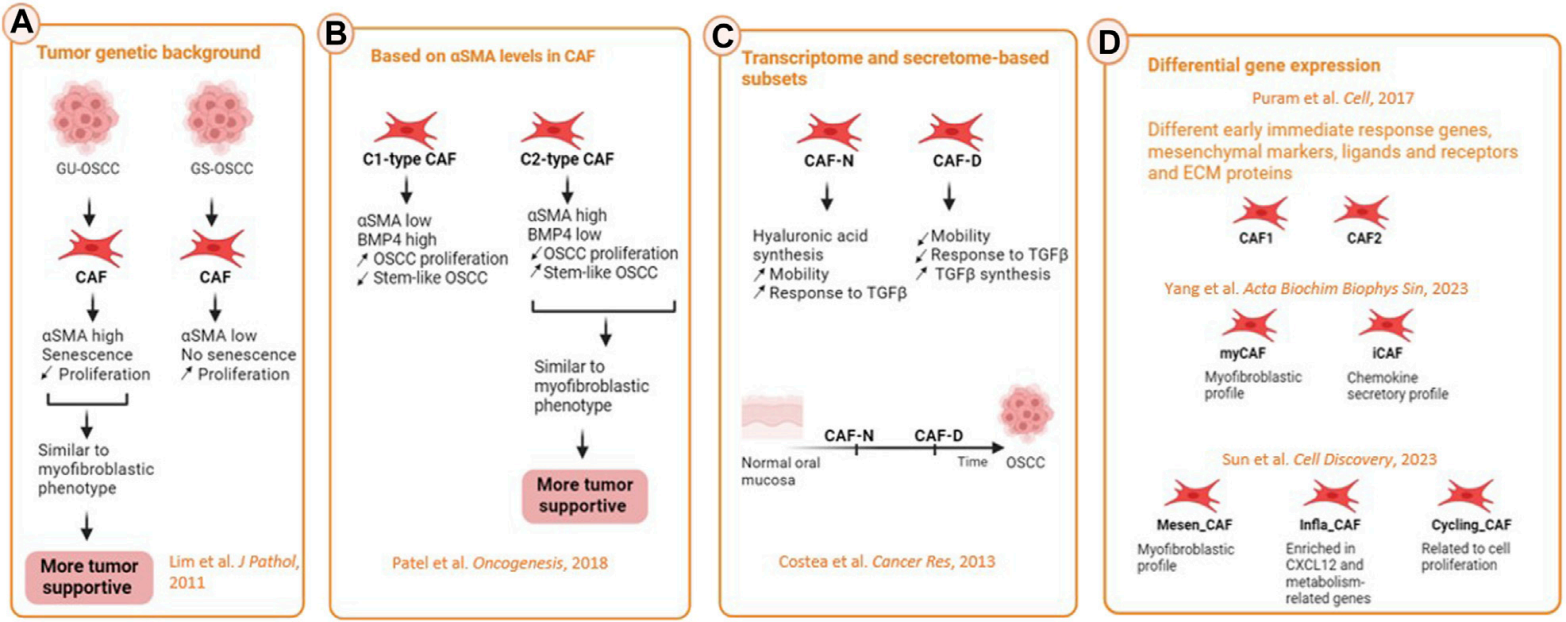
CAF heterogeneity in HNSCC. **(A)** Influence of the genetic background such as the status of p53 of the cancer cells on CAF heterogeneity. GU-OSCC were more similar to myofibroblastic phenotype because of their senescence and high expression of αSMA. CAF from this type of cancer are more tumor-supportive then the ones from GS-OSCC tumors. **(B)** On the basis of differentially secreted molecules, CAF could be separated in C1-type and C2-type CAF. C1-type expressing high αSMA and low BMP4 promoted the occurrence of cancer stem cells and were considered more tumor-supportive. **(C)** On the basis of the transcriptome, secretome, motile ability and response to TGFβ, CAF were differentiated into normal (CAF-N) and divergent (CAF-D). CAF-N secreted high levels of hyaluronan while CAF-D secreted high amounts of TGFβ, the former being more tumor-supportive. **(D)** Based on scRNA-seq, most of the studies identified at least one myCAF and iCAF subpopulation among different other CAF subtypes (Created with Biorender).

The existence of different CAF subtypes (Figure 3) with different functions has been first shown by functional studies (Lim et al., 2011; Costea et al., 2013). The genetic background of the tumor can shape CAF phenotype. Lim et al. (2011) compared CAF from OSCC lesions with wild type *TP53,* coined genetically stable OSCC (GS-OSCC) to CAF from OSCC lesions with mutated *TP53,* coined genetically unstable OSCC (GU-OSCC). Oral cancer cells derived from GU-OSCC tumors induced CAF senescence whereas genetically stable and dysplastic cells lacked this ability. Genetically unstable tumors upregulated αSMA expression and induction of senescence in oral fibroblasts which led them to a more myofibroblast phenotype. Senescent αSMA^high^ CAF proliferated less but were more tumor-supportive than non-senescent ones (Lim et al., 2011). We have also conducted a study investigating OSCC-derived CAF as compared to normal oral fibroblasts (NOF) through transcriptomic analysis of cells cultured in both 2D and 3D cultures. This investigation revealed two distinct populations of CAF. Specifically, the transcriptome and secretome of a certain subpopulation of CAF fibroblasts exhibited greater similarity to NOF and was thereby coined as CAF-N (normal-like) subtype. This similarity was also shown at the functional level, since CAF-N responded to TGFβ activation, similar NOF. In addition, the CAF-N phenotype displayed new, distinct features from NOF and the other CAF subtype such as enhanced intrinsic mobility sustained through increased autocrine hyaluronic acid synthesis. The second CAF subtype that we identified showed a more heterogenous transcriptome and therefore were named CAF- D (divergent). The cells that showed this phenotype were less motile and less responsive to TGFβ activation, while synthesizing much higher levels of TGFβ1^9^. Based on the spectrum of similarities to NOF, we proposed that the two CAF subpopulations may be a spectrum in the development of CAF, with CAF-N representing an earlier stage of differentiation.

More recently, a thorough characterisation of CAF subtypes in HNSCC has been done through (scRNA-seq. CAFCAFOne of the CAF subtypes identified was the myofibroblastic one (myCAF) (Puram et al., 2017). These were αSMA-positive contractile cells generated primarily by TGFβ signalling, whose main role being to secrete ECM. The second subset displayed ECM receptors, ligands, and genes, such as connective tissue growth factor (CTGF), PDPN, and FAP, all of which have been previously linked to the CAF phenotype. This second subset of CAF was further classified into two categories (CAF1 and CAF2) based on the differential expression of early immediate response genes, mesenchymal lineage markers, several signaling molecules and receptors, as well as proteins in ECM (Puram et al., 2017). The third category, which lacked myofibroblast and CAF markers, was suggested to represent fibroblasts that are dormant (Puram et al., 2017). By transcriptomic analysis on CAF cultures from OSCC, Patel et al. (2018) described two different types of CAF, C1-type CAF (αSMA^low^), which expressed greater amounts of BMP4 and were able to generate a higher proliferation of oral cancer cells and lower frequency of oral stem-like cancer cells; and C2-type CAF with a higher score of αSMAs (αSMA^high^), which exhibited lower BMP4 expression levels and induced lower proliferation of oral cancer cells with a higher frequency of stem-like cancer cells.

ScRNA-seq profiling of stepwise progressive lesions of HNSCC revealed three (Sun et al., 2023) and five (Choi et al., 2023) distinct CAF clusters with different transcriptomic profile. Sun et al. (2023) identified Mesen_CAF that expressed ECM related genes and mesenchymal-promoting transcription factors resembling to the myofibroblastic CAF (myCAF); Infla_CAF that were enriched in stromal-cell derived factor CXCL12, and metabolism-related markers; and Cycling_CAF that were related to cell proliferation. Choi et al. (2023) identified two CAF subpopulations that were similar to Puram’s CAF1, whereas two others showed a more myofibroblastic profile similar to Puram’s CAF2; and the fifth one expressed CXCL8, which is known to be involved in HNSCC progression. This CAF subtype was the last CAF subtype to appear in their pseudo-time analysis trajectory. Yang et al. (2022) that identified again an inflammatory subset (iCAF) and a myofibroblastic subset (mCAF) and showed that the proportion of each of these two CAF subtype changes according to the stage of progression of OSCC with an myCAF/iCAF ratio in favor of myCAF in the later stages of the cancerous lesions. Of note, one of the pathways upregulated in CAF in HNSCC has been shown to be the AKT3 pathway which promotes their myofibroblastic phenotype (Takahashi et al., 2021). However, despite progress in identifying several CAF subtypes in HNSCC, the characterization of CAF heterogeneity remains incomplete, and their functions remain unclear, their effect on HNSCC progression remaining an intriguing area of research.

## Tumor-promoting roles of CAF

In the advanced stages of HNSCC, CAF can constitute as much as 80% of the tumor mass and are commonly perceived to have a supportive role in promoting tumor growth (Dongre et al., 2022). Changes in the transcriptome, proteome, secretome and metabolome developed during conversion into CAF provide them with tumor-promoting abilities. The effect of CAF on various aspects of HNSCC cell plasticity are central in this aspect. CAF have a variety of ways in which they can increase HNSCC cell plasticity, either by increasing cancer cell stemness, EMT, and metabolic coupling, resulting ultimately in increased resistance to therapy and immune evasion. The mechanisms mediated by CAF have become a central area of stromal tumor research being proposed as novel therapeutic targets for the treatment of HNSCC.

### CAF-induced angiogenesis and proliferation

The paracrine factors secreted by CAF involve, among others, vascular endothelial growth factors (VEGFs), PDGFs, hepatocyte growth factor (HGF), and other chemokines and cytokines that may be responsible for tumor vascularization. Most prominent are the family of growth factors belonging to VEGFs (VEGF-A, VEGF-B, VEGF-C, and VEGF-D). Indeed, the neo-angiogenic VEGF-A and VEGF-B bind to their related receptor tyrosine kinases, VEGFR-1 and VEGFR-2 on the endothelial cells and drive their proliferation (Dongre et al., 2022). Furthermore, around one-third of oral tongue squamous cell carcinomas cases had CAF expressing neurogenic locus notch homolog protein 3 (NOTCH3), which was positively correlated with the size of the tumor and linked to an augmentation in microvessel density, indicating that Notch signalling in CAF could also promote tumor angiogenesis (Kayamori et al., 2016). Also, the CAF-induced matrix remodelling, carried by a series of matrix-metalloproteinases (MMPs), releases a huge amount of other growth factors sequestered in the ECM. Among them, the families of VEGFs and FGFs, are potent inducers of angiogenesis, making CAF a key regulator of tumor growth (Dongre et al., 2022).

CAF have been shown to promote cancer cell proliferation in multiple tumor types. Co-injection of HNSCC cells with CAF stimulated tumor growth *in vivo*, corroborating with the increased proliferation of HNSCC cells when treated *in vitro* with conditioned medium derived from CAF. Furthermore, several cytokines and growth factors synthesised by CAF have been shown to stimulate HNSCC cell and tumor growth, such as chemokine ligand 7, interleukin-8 (IL-8) and C-X-C motif chemokine 11 (Bae et al., 2014). In addition to CAF-SCC cells paracrine loops supporting OSCC cell proliferation, the ability of CAF to model the ECM through secretion of several collagens (such as collagen8A1 and collagen11A1) could also stimulate cancer cell growth through interaction with discoidin domain receptor 1 (Lai et al., 2019).

### Metabolic adaptation

Another aspect of the interplay between cancer cells and CAF is the metabolic coupling through which CAF support the high energetic demands of cancer cells. A phenomenon called “reverse Warburg effect” has been shown to occur in OSCC, in which CAF metabolism is switched towards aerobic glycolysis; the metabolic products (lactate and pyruvate) are then shuttled to OSCC cells and used by these cells in oxidative phosphorylation; in addition, mitochondria were also shown to be shuttled from fibroblasts to OSCC cells empowering them energetically (Zhang et al., 2020). Until now, the impact of the changes in the metabolic programmes of CAF has been studied mostly in the context of their influence on cancer cell growth, proliferation, invasion, and resistance to drugs. Not only may the metabolic reprogramming of CAF result from paracrine signaling from cancer cells but direct intercellular contacts between CAF and cancer cells may stimulate and reinforce mutual metabolic reprogramming. Glycolysis can also be favoured due to a preferred unidirectional transfer of mitochondria from fibroblasts to OSCC cells (Zhang et al., 2020). When the metabolism of stromal cells is subverted to undergo aerobic glycolysis, monocarboxylate transporters (MCT) are upregulated in both lactate-exporting cells (MCT4) and recipient cells (MCT1). OSCC cells were shown to upregulate MCT1 compared to normal oral keratinocytes, demonstrating their lactate-recipient role, while CAF were shown to upregulate the lactate-exporting transporter MCT4 compared to NOF. Lactate has been proven to have pro-tumor effects on its own, being involved in promoting angiogenesis, metastasis and generating an immune-suppressive microenvironment. Zhang et al. (2020) showed that OSCC cells were able to induce a metabolic reprogramming of NOF. After co-culture with OSCC cells, NOF were rapidly activated and acquired a CAF-metabolic phenotype by undergoing aerobic glycolysis, secreting high L-lactate, and overexpressing lactate exporter MCT4. Of importance, it was observed that the metabolic reprogramming with catabolite, mitochondrial transfer and mitophagy occurred even before the increased expression of αSMA and FAP in NOF. These data point to a certain sequence of tumor-fibroblast interaction events, in which the metabolic changes and reprogramming occur earlier before fibroblasts transition into a CAF phenotype (Zhang et al., 2020). Phosphofructokinase-2/fructose2,6-biphosphatase3 (PFKFB3) has been highlighted as a metabolic enzyme regulating glycometabolism in HNSCC. Inhibition of PFKFB3-dependent glycolysis altered the expression of proangiogenic factors (VEGF-A, PDGF-C and MMP9) in oral CAF. This highlighted a proangiogenic phenotype of HNSCC CAF regulated by glycometabolism (Li et al., 2022). Furthermore, Yang et al. (2019) showed that PFKFB3-dependent lymphotoxin-alpha promoted human umbilical vein endothelial cell proliferation and migration in HNSCC, which may contribute to aberrant angiogenesis. The PFKFB3-mediated glycolysis pathway has been described to be influenced by H19 long non-coding RNAs (lncRNA) on the reprogramming of glucose metabolism in oral CAF (Yang et al., 2019). LncRNAs are hypothesized to contribute to glucose metabolism by inhibiting the MAPK signalling pathway, PFKFB3, and miR-675-5p. Using RNA sequencing Yang et al. analysed lncRNA/mRNA profiles of NOF derived from normal tissues and CAF derived from patients with OSCC. LncRNA H19 knockdown affected proliferation, migration, and glycolysis in oral CAF. Using a luciferase reporter system, it has been shown that PFKFB3 is a target gene of the H19 lncRNA-derived miR-675-5p. In fact, miR-675-5p could affect the PFKFB3-mediated glycolysis pathway and reprogram the glycolysis state in the oral CAF (Yang et al., 2019). The symbiotic relationship between CAF and cancer cells creates a tumor metabolic ecosystem that could be a potential target for cancer therapy.

### EMT and metastasis

The increased plasticity and the partial epithelial-to-mesenchymal (p-EMT) program in HNSCC could be responsive to different CAF stimulation cues. Pal et al. investigated the *in situ* spatial localization of p-EMT program within HNSCC tumors; they found that paracrine interactions between CAF and malignant cells promote a p-EMT program at the leading edge of HNSCC tumors with potential roles in tumor invasion (Pal et al., 2021). While traditionally most of the research on invasion of cancer cells has been focused on metalloproteases and their important role in EMT degradation and local invasion, metalloprotease-independent mechanisms of cancer cell migration and invasion were also proven to occur in OSCC cell invasion, and CAF were major players in these mechanisms. By exerting contractile forces, CAF were proven to alter the organization and the physical properties of the basement membrane, making it permissive to cancer cell invasion (Pal et al., 2021). Gaggioli et al. demonstrated that CAF-induced invasion of adjacent OSCC cells is induced by CAF that are creating “tracks” in ECM through which OSCC cells invade, following CAF, which are naturally motile cells. CAF-generated tracks were dependent on α3 and α5 integrins and the activity of Rho and Rho-associated protein kinases. This study suggested that one mechanism through which OSCC invade locally is one dependent on the ability of CAF to remodel the ECM (Gaggioli et al., 2007).

CAF may also contribute to bone invasion of head and neck tumors. Notably, αSMA-positive CAF were commonly seen invading bone before cancer cells, and because they were more likely to induce osteoclastogenesis in macrophages than cancer cells, perhaps making them actors in the bone invasion in OSCC by controlling both macrophages and cancer cells. Primary human osteoblasts cultured with conditioned media of human OSCC-derived cells and primary human CAF showed a significant increase in receptor activator of nuclear factor kappa beta (RANKL) mRNA expression and a decrease in osteoprotegerin expression (Elmusrati et al., 2017). Bone resorption usually involves the activation of osteoclasts by the interaction of the RANKL. Osteoprotegerin is a decoy receptor for RANKL which can be expressed by osteoblastic cells, which prevents excessive bone resorption by preventing the interaction of RANK-RANKL. Both oral cancer and stromal cells secrete RANKL, but CAF have been shown to contribute to a greater extent to osteoclastic bone resorption *in vitro* and induced multinucleation of murine macrophages, these latter possibly behaving as osteoclasts (Elmusrati et al., 2017).

CAF are important mediators of tumor growth at the metastatic site by releasing growth factors and cytokines into circulation. They may emerge from the tumor sites or from the bone marrow and be recruited to metastases, or they may be activated tissue-resident fibroblasts as a result of metastatic cancer cell seeding and inflammatory responses. Within the metastatic niche, CAF promote the growth of secondary tumours. In the case of HNSCC, lymph node metastases involve high expression of c-Met (Raj et al., 2022). Paracrine activation of c-Met, caused by the secretion of HGF by CAF, facilitated the progression of HNSCC. In addition, vascular dissemination of OSCC cells and local invasion could be enhanced by focal adhesion kinase (FAK) signalling within CAF, and it has been shown that FAK knockdown in CAF could inhibit OSCC metastasis (Raj et al., 2022).

### CAF-related immunomodulation

In the latest period of immunotherapy development, the immunomodulation abilities of CAF draw a lot of attention in the CAF research field. It has been shown that CAF could protect tumors from immune attack by multiple mechanisms. In HNSCC, CAF affect immune cells both directly and indirectly. Networks of ECM proteins that CAF build are thought to prevent immune cells from accessing cancer cells, inferring that CAF and their generated ECM serve as a physical barrier to tumor infiltration by immune cells. CAF can interact with cells such as immune cells, including T cells and macrophages, and with cells that participate in bone remodelling (Thiery, 2022). Many studies have focused on CAF role in promoting resistance to antibody checkpoint inhibitors. Use of immune checkpoint inhibitors such as anti-programmed cell death protein 1 (PD-1) or anti-programmed death-ligand 1 (PD-L1), is already approved for clinical use for metastatic/recurrent HNSCC, but only one in five patients responds to this type of therapy (Vermorken et al., 2008), and much effort is currently placed to understand this and to identify biomarkers of response of immunotherapy in HNSCC.

Interesting to note is that in OSCC, pro-tumor M2 macrophages are the most prevalent immune cells around αSMA+, FSP1+, and FAP+ CAF-rich areas (Timperi and Romano, 2023). This suggests a close relationship between these two cell populations, which could have a significant impact on the clinical outcome for patients. CAF, either alone or cooperating synergistically with cancer cells, can skew the differentiation of monocytes to the pro-tumoral M2 macrophage phenotype. Then, these macrophages will exert suppressive effects on T cells, partly by secreting TGFβ, IL-10 and arginase (Takahashi et al., 2017). Once polarized to the M2 phenotype, macrophages will also affect oral cancer cells by enhancing growth, invasion, migration and cancer stem cell features. CAF-secreted chemokine (C-X-C motif) ligand 2 is involved in monocyte chemoattraction whereas interleukin-6 (IL-6) and granulocyte-macrophage colony-stimulating factor is involved in monocyte differentiation. Clinicopathological analysis has revealed a positive relationship between the abundance of CAF and tumour-associated macrophages in OSCC tumor samples, while both these cell types correlated with vascular invasion (Takahashi et al., 2017).

Furthermore, αSMA^+^FAP^+^ CAF from HNSCC, through IL-6 secretion, were shown to cooperate with TGFβ to inhibit CD8^+^ T cell proliferation and promote the recruitment of Tregs. On one side, CAF induced apoptosis of CD4 and CD8 T cells while increasing the proportion and migration of the Tregs that inhibit the T-cell antitumor response (Takahashi et al., 2015). CAF involvement in promoting an immune suppressive microenvironment has generated much interest in recent years, especially concerning resistance to immunotherapy targeting the PD-1/PD-L1 checkpoint. Takahashi et al. (2015) found that PD-L1 and PD-L2 are highly expressed by CAF in HNSCC and they interact with PD-1-expressing T-cells to suppress their effector function. CAF were also shown to suppress the infiltration of CD8 T-cells into tumors; in part, this is thought as a consequence of the deposition of a dense ECM by myCAF, which can act as a “barrier” to immune cells. Collagen, fibronectin, and different proteoglycans such as hyaluronan and versican, produced by myCAF, have been demonstrated to “trap” T-cells, hinder T-cell movement, and restrict T-cell infiltration into tumors (Evanko et al., 2012).

## CAF induced therapy-resistance

There is increasing evidence supporting a significant role for CAF in therapy-resistance in many solid cancers, including HNSCC. Mechanisms of resistance involving the stroma include the modulation of pathways involving cancer cell–ECM interactions, CAF–ECM adhesion and cytokine- or chemokine-mediated signalling pathways, or cancer stem cell like enhanced phenotype related to CAF-induced hypoxia. CAF have been demonstrated to provide resistance to many different cancer therapies, such as radiotherapy, chemotherapy (cisplatin), and immunotherapy (cetuximab, anti-PD-1/PD-L1 checkpoint inhibitors). This brought up the idea that targeting CAF therapeutically might enhance response rates across a broad spectrum of treatments.

Cisplatin promoted CAF survival after *in vitro* treatments and might induce oral fibroblasts to exhibit a CAF-senescent phenotype. Additionally, cisplatin promoted CAF-exosome biogenesis which appeared to have a unique composition since they could promote malignant characteristics in HNSCC (Qin et al., 2019).

Johansson et al. (2012) reported that ECM and CAF induced MMP-mediated cetuximab resistance in HNSCC. MMPs can be secreted by CAF to facilitate cancer progression in the context of angiogenesis, tumor growth, but also invasion by ECM remodelling. These results pinpointed to a novel CAF-dependent modulation of cetuximab sensitivity and suggested that MMP inhibition may enhance the effects of EGFR-targeted therapy. MMP1 silencing restored the response to cetuximab, confirming the importance of the right matrix rigidity for optimal response to molecular therapies.

A hypoxia CAF-induced phenotype has been also shown to lead to increased cancer growth, enhanced stem cell properties and resistance to chemotherapy (Xie et al., 2021).

Radiotherapy induces a healing response, promoting inflammation, and modulation of CAF and ECM remodelling, although CAF are considered relatively radioresistant. Kamochi et al. (2008) showed that after irradiation, fibroblasts upregulated TGFβ1 expression, underwent myofibroblast differentiation and increased the invasive growth of OSCC cells. Huang et al. (2021) showed higher amount of CAF in radioresistant nasopharyngeal carcinoma than the radiosensitive lesions which was modulated through IL-8/NF-κB signalling. Tranilast, a drug known to inhibit TGFβ signaling and fibrosis was shown to interfere with this mechanism (Huang et al., 2021).

## CAF-targeting strategies

**FIGURE 4:**
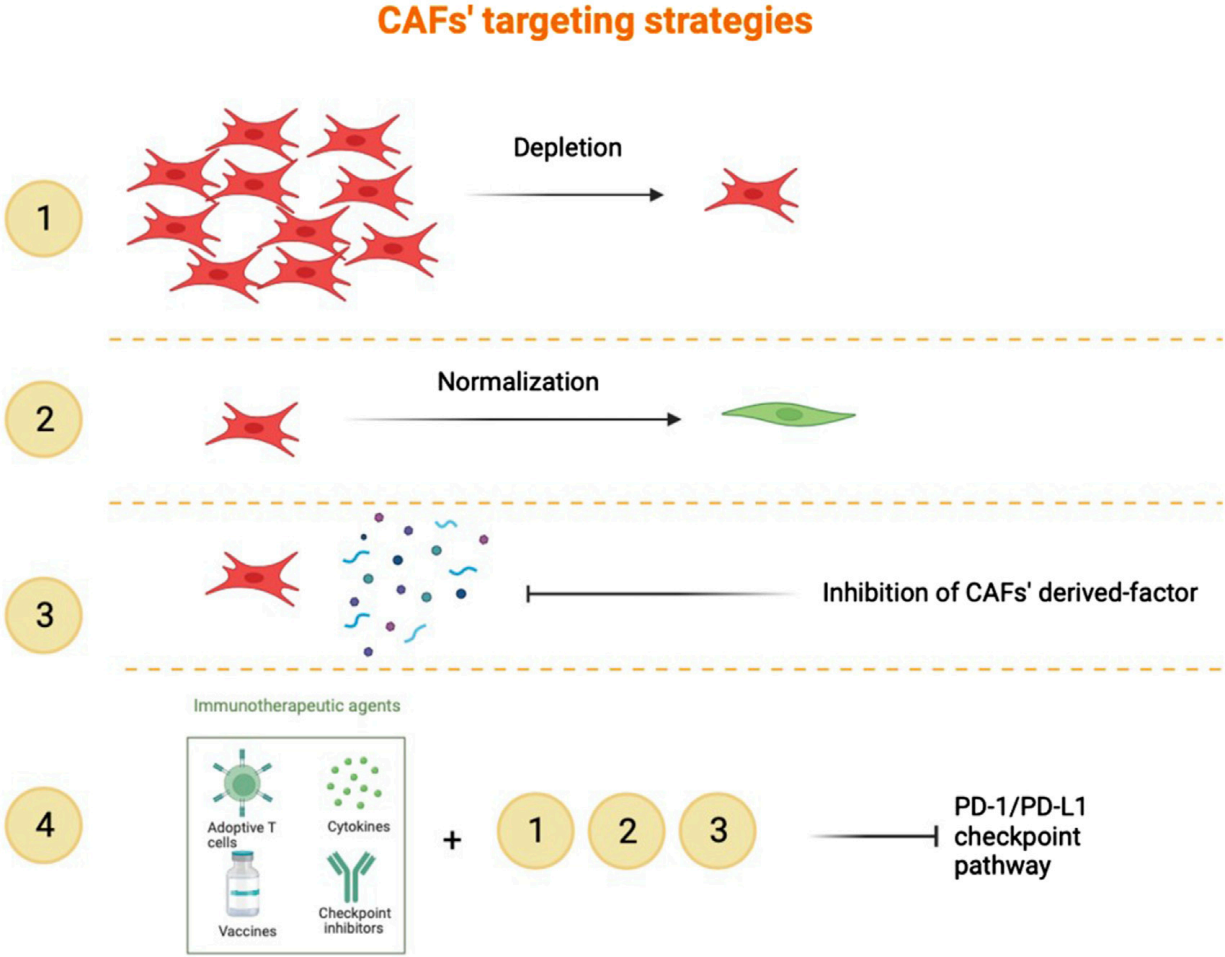
Targeting of CAF. 1). Depletion with a specific CAF marker to reduce the pro-tumour properties of CAF. 2). Normalization consists of an inhibition of the new activities acquired by CAF in the course of tumorigenesis. 3). Targeting the CAF secretome in order to inhibit its function and interactivity with other cells. 4). Combination of immune checkpoint inhibitors in addition to the strategies listed above.

Current treatment options for HNSCC include surgery, radiation therapy, chemotherapy, and more recently, treatment with anti-EGFR antibodies and checkpoint inhibitors for recurrent/metastasising HNSCC. Targeted immunotherapy has improved HNSCC patients’ survival, but less than 20% of patients produce a durable response to these treatments. CAF have emerged as key players in promoting cancer cell evasion of anticancer therapies. An ideal treatment would likely target both cancer and non-cancer compartments. Potential strategies for CAF-directed therapy include depleting CAF within the tumor microenvironment, “normalizing” CAF or inhibiting CAF activation or function (Figure 4). In order to do this, developing specific biomarkers to accurately identify CAF subtypes would be of crucial impact. Understanding of the molecular mechanisms specific for a certain CAF subset predominant in a certain HNSCC lesion would enable us to create personalized therapies. Our view is that combining CAF-targeting agents with existing treatments is the way forward to overcome resistance and improve outcomes, at least in a subset of HNSCC.

### CAF depletion

Targeting FAP-positive CAF in murine models has been shown to enhance anti-tumor immunity in several solid tumor models; a FAP-DNA vaccine induced CD8^+^ and CD4^+^ T-cells and synergised with other tumor antigen-specific DNA vaccines to enhance anti-tumor immunity (Duperret et al., 2018). However, FAP is widely expressed in different cell types and there is to date no CAF-specific target that could render this approach effective. However, non-selective elimination of CAF was found to result in a disease exacerbation (observed in a mouse model), suggesting that the functional heterogeneity of CAF in tumor microenvironment is of significance for clinical outcome and indicating the existence and importance of CAF subpopulations that can facilitate an anti-tumor response (Kalluri, 2016).

### CAF normalization

In their study, Hanley et al. (2018) pinpointed NADPH Oxidase 4 (NOX4) as a pivotal controller of myCAF in HNSCC. They discovered that the inhibition of NOX4 with Setanaxib, a drug designed for treating organ fibrosis, not only impaired myCAF activation but also led to the “normalization” of established myCAF. More recently, Ford et al. (2020) developed myCAF-rich murine tumor models to investigate how CAF influence the immune microenvironment and affect response to different immunotherapy modalities. In this context, it has been demonstrated that Setanaxib can be effectively utilized to overcome myCAF-induced T-cell exclusion from tumors, enhancing the efficacy of antitumor vaccination and anti-PD-1 immunotherapies (Ford et al., 2020). Moreover, there are other ways to regulate the CAF’ activation. In OSCC, miR-145 levels are elevated in CAF compared to NOF. miR-145 targets multiple players in the TGFβ signalling pathway. TGFβ1 upregulation of miR-145 inhibits myofibroblast transdifferentiation preventing pro-tumorigenic consequences in fibroblasts (Melling et al., 2018). This suggests that exogenous manipulation of miR-145 in the tumor microenvironment could reduce the pro-tumorigenic myofibroblastic character of CAF. TGF serves also as a tumor suppressor in the early stages of carcinogenesis and is crucial for tissue homeostasis, targeting it may be problematic.

### Inhibition of CAF derived-factors

TGFβ neutralization reduced myCAF phenotype development, promoting the formation of a fibroblast population characterized by a strong response to interferon and heightened immunomodulatory properties in breast cancer (Costa et al., 2018). These changes correlated with the development of productive anti-tumor immunity and greater efficacy of PD-1 immunotherapy. In OSCC 3D models, pre-treatment of CAF with TGFβ small molecule inhibitors induced decreased invasion of OSCC cells in the collagen gels (Costea et al., 2013).

### Combination of all CAF-targeting strategies with immunotherapy

High expression of the checkpoint molecule PD-1 on T-cells is related to the immunological profile of HNSCC, particularly HPV+ tumors (Lyford-Pike et al., 2013). Numerous immune and non-immune cells, including CAF and tumor cells, typically exhibit PD-L1 expression. This makes the PD-1/PD-L1 checkpoint pathway a good target for boosting anti-tumor responses to control and eliminate HNSCCs. This may be applied either alone or in combination with other approaches.

## Conclusion

There is substantial evidence that TME, and in particular CAF, affects progression and treatment response of HNSCC. The versatility of CAF has been related to a certain extent to their heterogeneity, and it has been shown to lead to different pro-tumorigenic functions of CAF in HNSCC progression. CAF can promote HNSCC through their secretome, stimulating HNSCC cell growth, EMT and inducing angiogenesis which provides nutrients for tumor growth. CAF alter also the metabolic microenvironment to suffice the energetic demand of cancer cells. In addition, CAF are able to remodel the ECM to create passages for the cancer cells, promoting local invasion and metastasis. Although CAF have different functions on their own and communicate with cancerous cells, it is important to acknowledge their interaction with other cells in the TME, notably the immune landscape. We have highlighted in this review the case of macrophages and T-cells, but in the context of HNSCC, which are cancers with a high immune infiltrate, other interactions can take place. Due to the treatment resistance that CAF can put in place, it is of relevance to co-target CAF and cancer cells, possibly harnessing stroma-induced synthetic lethality pathways. Efforts to target or reprogram specific subtypes of CAF might offer great potential for cancer treatment, which may bring greater clinical benefit to cancer patients. However, while significant progress has been made in understanding the role of CAF in HNSCC, there remain substantial gaps in knowledge, particularly regarding their heterogeneity and specific functions. Future research should aim to address these gaps by focusing on the classification, molecular mechanisms, and interactions of CAF within the tumor microenvironment. Developing targeted therapies that consider CAF diversity and leveraging combination treatment strategies could potentially improve outcomes for HNSCC patients. Enhanced diagnostic tools are also essential to accurately profile CAF and tailor treatments accordingly, ultimately advancing the clinical management of HNSCC.
